# Investigating the effects of TMS-related somatosensory inputs on TMS-evoked potentials provides evidence against significant interaction

**DOI:** 10.1038/s41598-026-37418-w

**Published:** 2026-01-30

**Authors:** Pedro C. Gordon, Johanna Metsomaa, Paolo Belardinelli, Ulf Ziemann

**Affiliations:** 1 Department of Neurology & Stroke, University of Tübingen, Tübingen, Germany; 2 Hertie Institute for Clinical Brain Research, University of Tübingen, Tübingen, Germany; 3 Department of Neuroscience and Biomedical Engineering, Aalto University, Espoo, Finland; 4 BioMag Laboratory, HUS Medical Imaging Center, Helsinki University Hospital, Helsinki University and Aalto University School of Science, Helsinki, Finland; 5 Center for Mind/Brain Sciences—CIMeC, University of Trento, Trento, I-38123 Italy

**Keywords:** Electroencephalography, Transcranial magnetic stimulation, Evoked potentials, Sham procedure, TMS-EEG, Motor cortex, Biological techniques, Neuroscience

## Abstract

The combination of transcranial magnetic stimulation (TMS) and electroencephalography (EEG) has emerged as a non-invasive technique to probe cortical responsivity. However, interpreting TMS–EEG data is challenging due to sensory inputs generated by TMS, which cause peripherally evoked potentials (PEPs) that overlap with TMS-evoked potentials (TEPs). These sensory inputs may also modulate the cortical response, potentially distorting TEPs. To address this and evaluate methods for reducing PEP contamination, we compared two sham designs: a “PEP saturation” method, which delivers high-intensity somatosensory stimuli in both sham and real TMS to saturate PEPs in both conditions, and a “PEP individualized matching stimulus intensity calibration” method, which individually adjusts stimulus intensity to match the PEP amplitude of real TMS. In both conditions, the PEPs from sham and real TMS conditions should match, enabling the subtraction of this confounder. If the TEPs after this subtraction were not different between the two conditions this would indicate the absence of a relevant interaction between PEPs and TEPs, justifying the removal of PEPs from TEPs by subtraction. Our results showed no significant difference in TEPs within 110 ms post-stimulation after sham subtraction regardless of the sham protocol, and whether stimulating the primary motor cortex or the supplementary motor area. These findings provide evidence for the absence of a relevant interaction between TMS-related somatosensory input and TEPs, and indicate the appropriateness of the two sham protocols in removing PEPs from the TMS-EEG response.

**Supplementary Information:**

The online version contains supplementary material available at 10.1038/s41598-026-37418-w.

All subjects provided written informed consent prior to screening and measurements. The study was approved by the ethics committee of the medical faculty of the University of Tübingen (810/2021BO2), following the latest version of the Declaration of Helsinki.

## Introduction

The combined use of electroencephalography (EEG) and transcranial magnetic stimulation (TMS) is under development since the late 1990s as a non-invasive method to probe cortical responsiveness to electromagnetic stimuli^1–3^. TMS-EEG has been applied in various domains, such as studying the effects of drugs on the human central nervous system, or identifying diagnostic and prognostic markers of brain diseases, such as disorders of consciousness^2,4^. However, substantial debate remains regarding the interpretation of TMS–EEG data^5,6^. One primary issue concerns the cortical responses to sensory inputs generated by the TMS, including auditory stimuli from the high-pitched “click” sound generated during coil discharge^7^ and somatosensory stimuli from the activation of scalp nerve endings and cranial muscles near the TMS site^8^.

There is significant evidence suggesting that cortical responses to sensory inputs—known as peripherally evoked potentials (PEPs)—overlap with cortical responses to direct cortical activation induced by TMS, the TMS-evoked potentials (TEPs)^6,8–14^. PEPs typically emerge approximately 70 ms after the TMS pulse (although earlier responses may also be observed) and include a negative deflection at around 100 ms followed by a positive deflection at around 200 ms, predominantly in frontocentral regions^10,11,15^. Although previous TMS-EEG studies named these responses based on a specific sensory modality, such as auditory evoked potentials or somatosensory evoked potentials, PEPs are not solely elicited by auditory or somatosensory inputs but reflect a multimodal response to incoming sensory stimuli, as well as top-down attentional processing^16,17^. The presence of PEPs in TMS–EEG data poses a challenge, as these sensory responses may be misinterpreted as TEPs, which are exclusively responses to TMS-induced cortical activation. This issue is particularly problematic when using TMS–EEG to investigate changes in cortical function, such as in neuropsychiatric diseases or in response to interventions that modulate cortical responsiveness. Several neuropsychiatric disorders and intervention are known to alter sensory processing and consequently PEPs^18–20^, and this in turn may confound TMS-EEG results, leading to inaccurate interpretation of TEP data, limiting the method’s utility^21^.

To address auditory input, one proposed solution is to deliver masking noise through headphones during TMS–EEG measurements, preventing subjects from perceiving the TMS click sound and thereby blocking auditory event-related potentials^22^. Although this approach has been mostly successful, some studies report that it does not fully mask the TMS click sound, especially when high TMS intensities are used^8,13^. Addressing somatosensory input is even more challenging, as no successful method for masking this sensation has been reported to date. One potential solution involves using a control condition with sham TMS, which simulates the sensory inputs of real TMS without directly stimulating the cortex. In this design, the sensory responses from both sham and real TMS are attributed to PEPs, while components observed exclusively with real TMS are considered TEPs^12,13,23,24^. Additionally, adding a click sound via a sham TMS coil can simulate the auditory input from real TMS, thus addressing the limitations of the masking noise approach.

However, this solution comes with its own challenges. The responses evoked by the sham procedure may not perfectly match those from real TMS due to the different nature of the somatosensory stimuli. Specifically, the sensory input from sham TMS (electric current) and real TMS (induced electromagnetic field) activate different sensory nerve endings, making precise replication of the sensory experience difficult. To address this issue, we have proposed using high-intensity somatosensory inputs for both sham and real TMS conditions, rather than trying to replicate the exact sensory input from real TMS^13^. By delivering a more intense sensory stimulus than typically expected from real TMS, both sham and real TMS conditions lead to saturation of PEP amplitude. As a result, any additional sensory input from real TMS will cause no or only minimal changes in PEP amplitude, leading to comparable PEPs across both conditions. These PEPs can then be subtracted from the real TMS response, thus isolating the TEPs.

While this method has been effective in eliminating the PEP component and revealing TEPs in TMS–EEG experiments^13,21^, it relies on the assumption that TEPs are not modulated by sensory input. Given that the sensory response is intentionally saturated, this assumption may not hold, as the intensity of sensory input used in this method is much higher than in previous TMS–EEG studies. We previously tested this assumption in a pilot study, which found no evidence of TEP modulation by sensory inputs when stimulating the primary motor cortex (M1)^25^. The objectives of the present study are twofold. First, we sought to expand on the pilot findings by increasing the sample size and by including an additional cortical target: the supplementary motor area (SMA). Demonstrating either the presence or absence of sensory modulation of TEPs in responses originating from another cortical region, one known to produce distinct activation patterns, would serve as a conceptual replication and strengthen the validity of the conclusions. For this, our experiment involved the comparison of TEPs from TMS delivered with concomitant scalp electric stimulation (ES) at increasing intensities, using our “PEP saturation” sham design. Second, to more clearly determine whether sensory inputs modulate TEPs, it was essential to include a sham procedure with no accompanying additional somatosensory stimulation in the real TMS, but with sham stimuli that would elicit PEPs that reliably match those from real TMS. Designing an optimized realistic sham that met these criteria therefore constituted the second objective of the study.

If TMS–EEG responses from direct cortical activation are modulated by sensory stimulation, we expect TEPs to differ depending on the presence and the intensity of the concomitant sensory stimulus. Moreover, the differences should appear in the specific TMS-EEG responses from both M1 and SMA. Conversely, if TMS-EEG responses from direct cortical activation are not modulated by sensory stimulation, then the TEPs will not differ after subtraction of the sham from the real TMS-EEG response, irrespective of the presence and intensity of somatosensory stimulation. Finally, in this case the TEPs across all conditions should exhibit statistically significant similarities, as demonstrated by equivalence testing.

## Methods

### Subjects and design

A total of 20 participants (11 female) were included in this study, with a mean age of 26 years (SD ± 5.2 years). All subjects provided written informed consent prior to screening and measurements. The study was approved by the ethics committee of the medical faculty of the University of Tübingen (810/2021BO2), following the latest version of the Declaration of Helsinki.

The experiment involved right-handed healthy volunteers who participated in a single session. Inclusion criteria were age between 18 and 50 years and competence to provide informed consent to participate in the experiment. Subjects were excluded if presented with history of psychiatric or neurological diseases, current intake of drugs acting on the central nervous system, history of alcohol or illicit drugs abuse, or pregnancy.

### Experimental set-up

Prior to the TMS–EEG session all participants underwent magnetic resonance imaging (MRI) in a 3T Siemens PRISMA scanner for T1-weighted anatomical sequences. MRI was used for guiding TMS coil placement with respect to the individual’s brain anatomy by using a neuronavigation system (Localite GmbH, Sankt Augustin, Germany). Individual MR images were also used for EEG forward modeling and source reconstruction.

The TMS–EEG session was conducted with the participants sitting comfortably on a reclined chair and instructed to keep their eyes open during the measurements. Scalp EEG was recorded using a TMS compatible 64-channel Ag/AgCl sintered ring electrode cap (EasyCap GmbH, Germany) and EEG amplifiers (Bittium NeurOne, Finland). The EEG reference was placed in the CPz position and the ground in PPO1h. Surface EMG was recorded with adhesive hydrogel electrodes (Kendall, Covidien) over the abductor pollicis brevis (APB) and first dorsal interosseus (FDI) muscles of the right hand in a bipolar belly-tendon montage. The system’s sampling rate was 5 kHz. EMG was used to determine the resting motor threshold (RMT) and to record MEPs throughout the TMS–EEG measurements. Trials in which MEPs were detected were excluded from the TMS–EEG analysis. To establish the RMT, we first identified the cortical site that evoked the largest MEP amplitudes while the coil was oriented perpendicular to the central sulcus; this site was defined as the *hotspot*^26^. The RMT was then determined as the lowest stimulation intensity that elicited MEPs of at least 50 µV in ≥ 5/10 trials. In this study, the target muscle (either APB or FDI) was chosen based on which produced the larger MEP amplitudes. This approach was motivated by our aim to deliver subthreshold stimuli to M1 during the TMS-EEG measurements, thereby avoiding the generation of MEPs. If elicited, MEPs could produce reafferent cortical activity and further confound TEPs^27–29^. The procedure thus served to minimize the likelihood of motor responses during the experiment.

TMS was delivered by a figure-of-eight coil (external diameter of each wing, 90 mm) connected to a Magstim 200² magnetic stimulator (Magstim Company Ltd., UK), generating a monophasic current waveform. Two identical stimulators and coils were used in this experiment, one for real TMS and the other for sham TMS. As TMS targets, we selected the hand area of the left primary motor cortex (M1), i.e., the identified *hot-spot*, and the left supplementary motor area (SMA), identified in the individual MRI by MNI coordinates [-2, -7, 55]^30^. During TMS–EEG measurements, coil orientation was maintained perpendicular to the gyrus corresponding to the target, as this angulation was observed to elicit more effective cortical responses^31^. In M1, this corresponded to a posterior-to-anterior induced current, and in the left SMA to a right-to-left induced current. Proper coil positioning during all measurements was ensured using neuronavigation. Throughout all sham conditions, the intensity used for real TMS was 90% RMT when targeting M1, and 120% RMT when targeting the SMA. Effective neuronal activation in the cortex is achieved at TMS intensities higher than the RMT, as evidenced by intracranial recordings^32,33^. Moreover, usually higher TMS intensities are necessary to effectively activate frontal cortical regions compared to other regions^34^, which is why we selected a suprathreshold intensity for SMA stimulation. However, suprathreshold intensity stimulation to M1 elicits MEPs (by definition), which can in turn elicit reafference feedback responses in the EEG, as mentioned earlier. For this reason, we chose a subthreshold intensity for M1 stimulation. Additionally, in both cases we excluded trials that elicited MEPs, identified by the surface EMG recording during data processing.

During all measurements, subjects received masking noise delivered by earbuds. The masking noise was designed to have the same spectral distribution as the discharge click sound produced by the TMS coil used in the experiment, created with the „TMS Adaptable Auditory Control“ tool^22^. The noise intensity was individually set to be sufficient to suppress the coil click perception, or to maximum tolerable intensity. In addition, all sham conditions also included auditory sham on top of the masking noise, as will be detailed below. Electric stimulation (ES) of the scalp was delivered by a Digitimer DS7A (Digitimer Ltd. UK).

### General experimental design

TMS–EEG measurements were divided into 8 blocks, with 4 blocks involving TMS targeting M1, and 4 blocks targeting SMA. The 4 blocks of each TMS target corresponded to different sham procedures and, consequently, different nature and intensity of the concomitant sensory stimulus. The sham designs will be detailed in the next section. In each block 280 trials were obtained, 140 real TMS and 140 sham TMS trials, randomly interleaved, with a mean interstimulus interval of 2.5 s and a 0.5 s jitter. In total, 2 targets (M1, SMA) x 4 sham conditions x 2 stimulation types (real TMS, sham TMS) x 140 trials = 2240 trials were obtained per experiment. The order in which the blocks were delivered was randomized.

### Sham TMS design

**Fig. 1:**
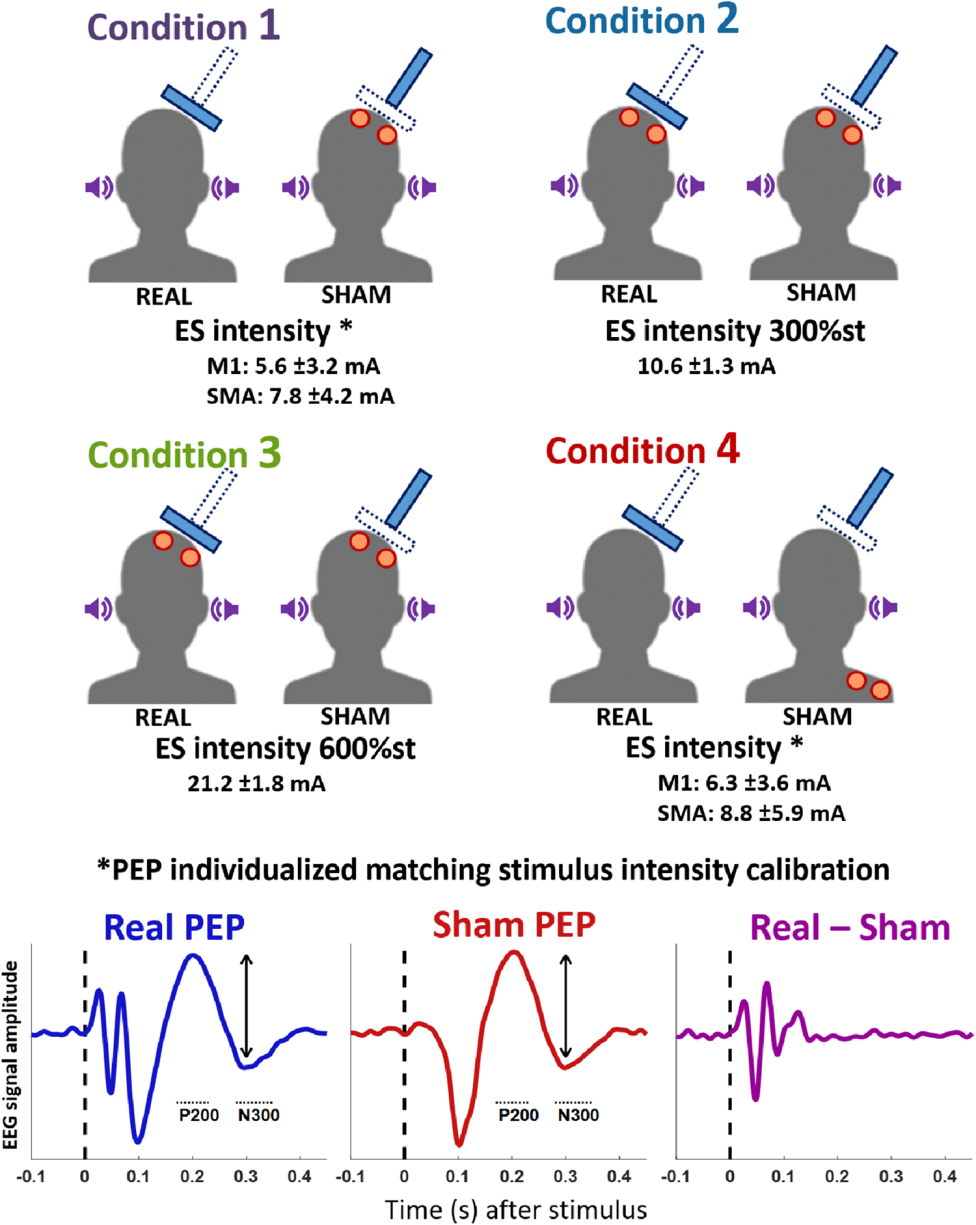
Illustration of the conditions in the present experiment. All conditions involved the delivery of masking noise (purple loudspeaker icon), skin electrical stimulation (ES) delivered by surface electrodes (red circles), and TMS pulses (bold blue rectangles). In the real TMS conditions, the TMS pulse was delivered with the coil properly tangential to the scalp, whereas in the sham conditions, the TMS was delivered with the coil perpendicular to the scalp. The conditions differed on the nature of the ES, which was either delivered on the left scalp (conditions 1, 2, 3) or on the left shoulder (condition 4). The ES intensity was either set as a proportion of the individual’s somatosensory perception threshold (conditions 2, 3; “PEP saturation” sham, in which case the ES was delivered during both the real and sham TMS), or ES intensity was individually calibrated to elicit a PEP of the same amplitude as from real TMS (conditions 1, 4; “PEP individualized matching stimulus intensity calibration” sham design (PIMSIC), in which case the ES was delivered only during the sham condition). Below each condition is displayed the mean ES intensity (±SD). Note that for the PIMSIC conditions, the intensity depends on the cortical target region.

In the present experiment we applied two different sham TMS methods, both involving the delivery of auditory input by a sham coil and somatosensory input by skin surface ES. In the “PEP individualized matching stimulus intensity calibration” design (PIMSIC), we individually calibrated the ES intensity, so that the PEPs had approximately the same amplitude as those from real TMS^25^. This was done by online visualization of the average of the EEG response to stimuli, enabled by the NeuroOne EEG system interface, and inspired by proposals to visualize TEPs in real-time^35^. Before the proper TMS–EEG measurements, we applied 40 single TMS pulses each to the two TMS targets (M1 and SMA), with masking noise on, and obtained the average response from 30 trials. The first 10 trials were excluded to allow for stimulus habituation^36^. We then estimated the PEP amplitude, which we defined as the peak-to-peak difference between the highest amplitude between 170 and 230 ms and the lowest amplitude between 280 and 350 ms, in the response from the FCz channel. We deliberately chose to avoid the involvement of the negative potential around 100 ms (N100) in this estimation because, despite the consistent association of this potential with sensory responses, there is evidence that TEP components overlap with the N100^13,37^. If we had calibrated the sham intensity to match a response that partially includes a TEP component, the result will be an overcorrection. Despite some preliminary evidence that TMS-evoked responses can be detected by intracranial recordings in time windows up to 400 ms after the pulse^38,39^, there is little evidence that scalp EEG deflections beyond 150 ms after the pulse can be due to TEPs^8,10,11,13,40,41^, thus justifying our choice. We then applied ES with varying intensities until we found a stimulation intensity that elicited PEPs of the same amplitude as those elicited by the real TMS, in the average of 30 trials and allowing for an error of ± 0.5µV (with a procedure duration of approximately 10–15 min). This corresponds to the “Condition 1”, with ES applied to the scalp, and “Condition 4”, with ES applied to the ipsilateral shoulder (Fig. 1). Note that the PIMSIC design did not involve ES in the real TMS conditions.

As the sensory perception, and consequently the PEPs, might change with the target for real TMS and the ES electrodes placement, individual intensity calibration was performed for each of the following conditions: M1-TMS with scalp ES, M1-TMS with shoulder ES, SMA-TMS with scalp ES, and SMA-TMS with shoulder ES. The average ES intensity applied in each condition is displayed in Fig. 1.

The second sham method involved the “PEP saturation” design, with ES intensity set according to the individual somatosensory perception threshold (i.e., the lowest intensity that can be perceived by the individual). Saturation of PEP amplitude occurred at around 300% of the somatosensory perception threshold with the present scalp electrode array set-up^13^. Therefore, “Condition 2” corresponds to the “PEP saturation” sham at 300% of the somatosensory perception threshold. Lastly, in “Condition 3” an intensity of 600% of the somatosensory perception threshold was used, corresponding to double the intensity of “Condition 2” in the attempt to cause a significantly different modulation of TEPs (Fig. 1). Importantly, by the “PEP saturation” method, ES was applied during both the sham and real TMS conditions.

In all conditions, ES was delivered by 2 pairs of electrodes of 1 cm in diameter. For scalp ES, electrodes were placed between the EEG electrodes, having 2 electrodes of the same polarity at FCC2h and TPP7h, and 2 of the other polarity at CPP5h and CCP2h. Polarity was inverted after each pulse to avoid charge build-up. This array differs from our previous report^13^, as in that study the broad area between different polarities was likely responsible for a considerable artifact in the early response signal. This change, however, required the use of longer pulse widths (100 µs for scalp ES and 200 µs for shoulder ES), which have lower perception thresholds. On the shoulder, 4 electrodes were placed in a square array, 3 cm apart, around the left acromion.

All sham conditions included the delivery of auditory sham on top of the masking noise, produced by the discharge of a second TMS coil mounted on top of the real TMS coil and angulated at 90 degrees (Fig. 1). Sham TMS intensity was set at 150% RMT. This was done to approximately match the auditory inputs between sham and real TMS conditions at the level of the outer ear canal^13^, to address the eventuality of the masking noise not being fully effective.

Plots at the bottom illustrate the PEP amplitude estimation from real TMS, considering the peak-to-peak amplitude difference between the peaks at around 200 ms and 300 ms (indicated by dotted lines labeled respectively P200 and N300), followed by calibration of the sham ES intensity until the PEP amplitude matched that in the real TMS condition. The subtraction of the sham response from the real TMS response (Real – Sham) should then reveal the “true” TEPs. The plots do not correspond to real data and are only meant for illustrating the procedure.

### EEG data processing

Offline data analysis was performed using the FieldTrip open source toolbox^42^ as well as custom-made Matlab functions. EEG data from TMS responses were segmented into epochs aligned to the TMS pulse (− 1500 to 1500 ms) and baseline corrected (− 500 to − 25 ms). Despite the optimized positioning of the ES array on the scalp, there was still a marked decay artifact observable in the first tens of milliseconds after the pulse in most datasets. To remove this artifact, we subtracted the best fit of an exponential function from each trial and channel^8^. For this purpose, we used the ‘fit’ function from MATLAB to fit the one-term exponential model (a*exp(b*x)) over every epoch during the time window 15 to 500 ms after the TMS pulse. With the prior knowledge that the decay artifact mostly affects early time points, a weighted fitting of the exponential model was used, which incorporated a linearly spaced array of weighting factors starting from 100 and ending at 1 over the course of the fitting time interval. Following the exponential decay subtraction, the time window between − 5 and 20 ms around the TMS pulse, still containing the high amplitude TMS artifact, was removed and cubic interpolated. EEG data were then downsampled to 1 kHz.

Trials were visually inspected, and epochs and channels with excessive noise were excluded, as were trials containing MEPs in the EMG of the right FDI or APB. On average, 3 channels (minimum 0, maximum 10 channels) and 15.5% of trials (± 10.7% SD) were excluded per measurement due to excessive noise. On average 5.8% of trials from real TMS contained MEPs (± 1.8% SD) and were excluded. In order to remove remaining TMS–EEG artifacts, we used the SOUND-SSP–SIR approach. The SOUND-SSP–SIR joint algorithm estimates the signal subspace containing the TMS-related artifacts and suppresses them from EEG signals^43–45^, Moreover, FastICA was applied to identify eye movement (blinks and saccades) topographies. Since their removal may spatially spread channel-wise noise, hampering the SOUND estimation process, the ocular artifacts were removed only after the SOUND-SSP–SIR step by beamforming^43,46^. Finally, for each subject, TMS-EEG responses of sham vs. real TMS trials were averaged, separately in conditions 1–4.

The resulting signals were also inverse-estimated into the source space, using the signal output from the SOUND-SSP–SIR and prior to the trial averaging. To this end, individual cortical surfaces and dipole arrays (a total number of 15684 source dipoles) were obtained from the individual’s MRI scans, which were segmented and meshed using the FieldTrip and FreeSurfer toolboxes^42,47^. The source space, in the form of the mid-thickness mesh as a geometrical average of grey matter and white matter boundaries, had each point adjusted for sulci and gyri on a spherical space. In this way, the individual points on the source space could be considered homologous across subjects. A customized pipeline was used for the Boundary Element Method EEG forward model, taking into account the positions of the EEG electrodes related to individual head anatomy, using a normalized lead-field^48^. Source reconstruction was then obtained on the whole cortical surface using the L2-minimum-norm estimate^49^. The result was standardized by z-transforming the signal of each trial with respect to the mean and standard deviation of the signal, yielding the signal of the 15,684 sources located on the mid-thickness mesh between grey and white matter. For meaningful statistical analysis we aimed at decreasing the degrees of freedom of the data by averaging the dipoles signals within predefined parcels, using the Glasser parcellation^50^. After removing parcels situated in deep structures, thus less likely to contribute to the overall EEG, we obtained 290 averaged source time-courses.

### Statistics

All statistical analyses were performed on the MATLAB platform (R2022a, The Mathworks, USA), comparing EEG responses using cluster-based and appropriate FieldTrip functions^42^. Firstly, we compared the EEG responses from the real TMS with the responses from the sham TMS for each condition, with trials from each condition averaged for each subject and comparisons performed pairwise in dependent samples t-tests on the subjects’ level. This involved conducting a cluster-based t-statistics to determine the time window (between 20 and 300 ms after the TMS) that contained significantly different EEG responses, including all channels and time samples^51^.

The EEG responses were then averaged within the significant time windows and compared by only including the channels in the cluster calculation, that yielded the significant channel clusters. The significance threshold was set to *p* < 0.05. A second analysis involved comparing the EEG response cleared from the PEPs (Real minus Sham response), which would correspond solely to TEPs, across the 4 conditions. For this purpose, the EEG response from the sham TMS was subtracted from the real TMS in each of the 4 conditions. The resulting EEG responses from the 4 conditions were then compared using cluster-based ANOVA to determine the time windows (between 20 and 300 ms after the TMS pulse) that contained significantly different signals. This was followed by post hoc cluster-based t-tests averaging the signal in the time windows where the ANOVA indicated a significant difference.

To better estimate the cortical location of the signal differences, we repeated the statistical analyses in the source space. In order to perform the cluster-based statistics in the source space, we designed a customized „neighbor layout“ as an input to the cluster-based t-test and ANOVA functions, which considered each of the 290 parcels as a „channel“ and the corresponding immediately surrounding parcels as its neighbors. It was required that multiple neighboring regions (set as a minimum of 3) had a t-value that exceeded the significance threshold to be included in the clustering algorithm.

Although the tests described above effectively detect differences in response signals, it is also essential to demonstrate that signals across conditions are statistically equivalent. To this end, we conducted equivalence testing using a bootstrapped resampling approach to estimate mean differences between TEP amplitudes and assess whether they fell within a predefined Region of Practical Equivalence (ROPE). If the mean differences between the TEPs yielded by the different conditions lie within the ROPE, the conditions are considered statistically equivalent. This procedure is conceptually similar to the Two One-Sided *t*-tests (TOST) method but it accommodates non-parametric distributions and comparisons across multiple groups^52,53^. Specifically, concerning the spatial domain, for each subject and condition, response amplitudes were averaged across all trials within predefined time windows based on observed evoked potentials: 30–50 ms, 50–80 ms, 80–120 ms, and 120–200 ms. A bootstrapping procedure (10,000 permutations) was then applied by resampling across all 20 subjects, calculating the mean differences across the four conditions for each permutation, along with the corresponding standard deviation. ROPE bounds were defined to reflect a medium or smaller effect size (Cohen’s d < 0.4). The resulting distribution of mean differences was then compared to the ROPE, and statistical equivalence was inferred if more than 97.5% of the distribution lay within the ROPE. This implies that only signal amplitude differences smaller than half a standard deviation would be considered significantly equivalent^54^. Additionally, for equivalence in the time domain, whole time-course analysis was performed by averaging signals within a cortical region of interest (corresponding to the TMS target) across all time points from 20 to 300 ms post-stimulation. Equivalence testing was then applied as described above, identifying time windows where signal amplitudes were significantly equivalent between conditions.

## Results

### Real vs. Sham TMS of M1

**Fig. 2:**
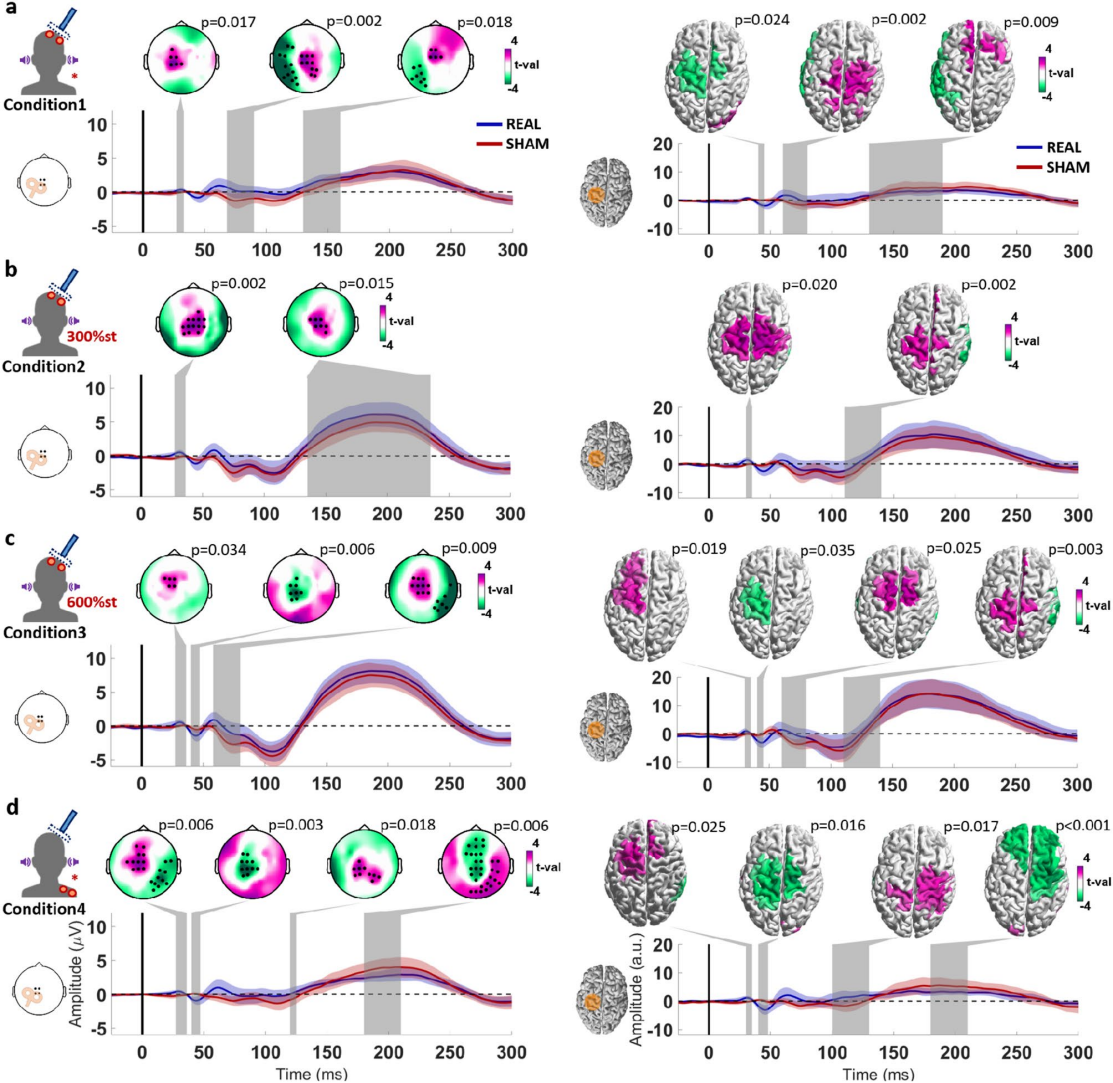
Statistical comparison of the TMS-EEG responses from real and sham TMS targeting the left M1, divided by each condition: **a-d.** Conditions 1–4 as described in Fig. 1. The topographical plots display the results from the cluster-based t-test, with electrodes composing significant clusters marked as black dots. The cortical models to the right show the results from the cluster-based t-test applied to the source reconstruction, with parcels composing the significant clusters colored by the respective t-values. The time window corresponding to each significant cluster is marked by a shaded gray area on each respective time course plot below. Time course plots on the left show the averaged signal from the electrodes FCz, FC1, Cz, C1 illustrated in the corresponding inlet to the left (the selected electrodes constitute the intersection between the significant clusters of the corresponding conditions); and on the right, averaged from the dipoles in the area marked in orange in the cortical model on the left.

Real and sham TMS of M1 elicited a high-amplitude negative deflection at around 100 ms after stimulation, followed by a positive deflection at around 200 ms, centered in the frontocentral cortex, in all conditions (Fig. 2 and Supplement Figure S1). The pattern is typical of the N100/P200 PEP complex. Also in line with PEPs are the higher amplitudes seen in conditions 2 and 3, which involve high-intensity ES to the scalp. The high-intensity ES conditions also reveal a positive deflection at around 50 ms, in line with the notion that sensory evoked responses might also be observed at this latency in TMS–EEG measurements^8^. Also, in the time window < 70 ms after stimulation, multiple signal deflections can be observed over the stimulated sensorimotor cortex after real TMS, but not after sham TMS, likely corresponding to the P30, N45 and P60 TEP components. However, in the high-intensity ES conditions 2 and 3, the P60 is partially obscured by a negative potential centered on the contralateral sensorimotor cortex, likely corresponding to the sensory evoked response.

**Fig. 3:**
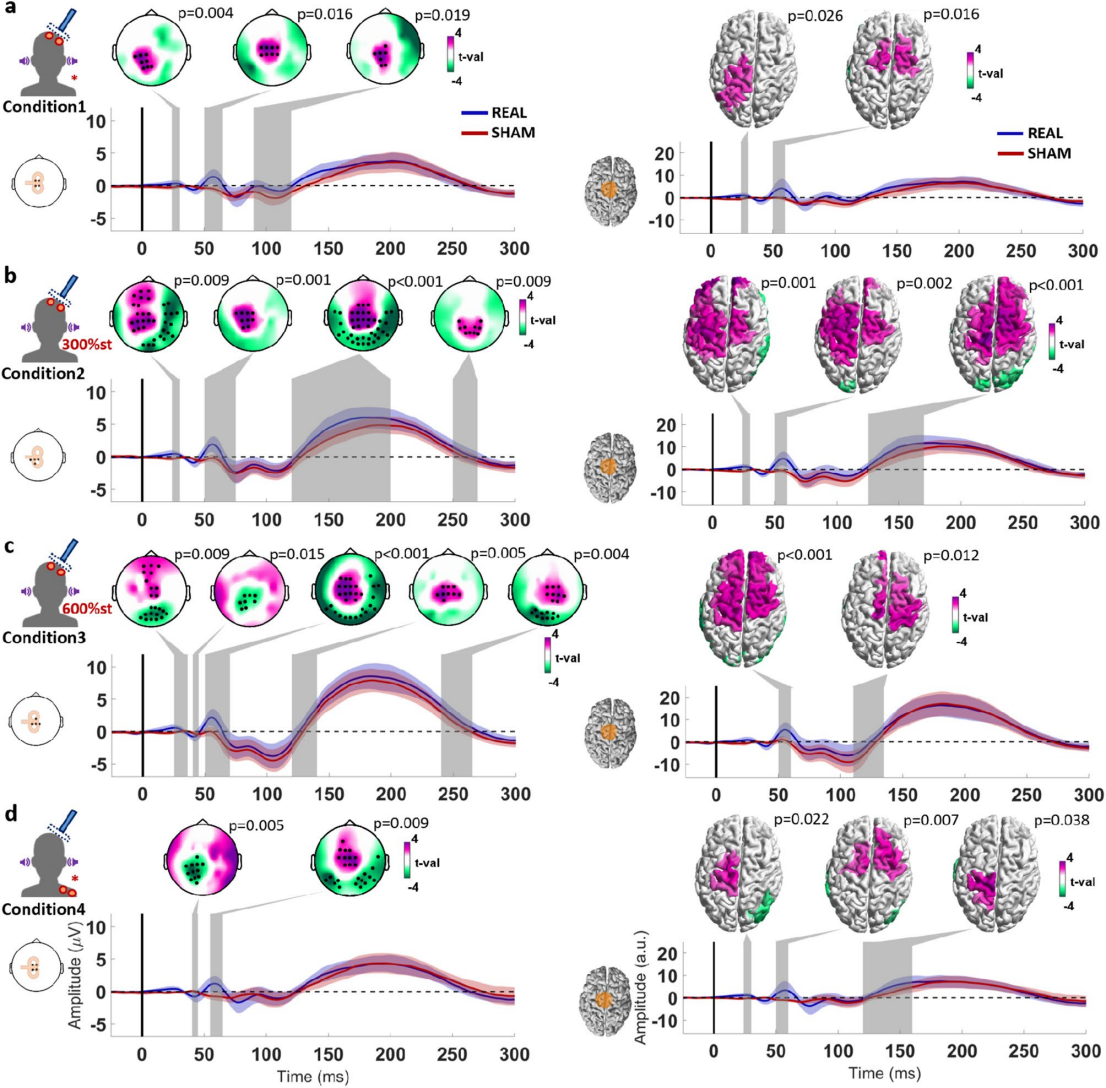
Statistical comparison of the TMS-EEG responses from real and sham TMS targeting the left SMA, divided by each condition: **a-d.** Conditions 1–4 as described in Fig. 1. The topographical plots display the results from the cluster-based t-test, with electrodes composing significant clusters marked as black dots. The cortical models to the right show the results from the cluster-based t-test applied to the source reconstruction, with parcels composing the significant clusters colored by the respective t-values. The time window corresponding to each significant cluster is marked by a shaded gray area on each respective time course plot below. Time course plots on the left show the averaged signal from the electrodes illustrated in the corresponding inlet to the left (the selected electrodes constitute the intersection between the significant clusters of the corresponding conditions); and on the right, averaged from the dipoles in the area marked in orange in the cortical model on the left. The shaded areas around the time course signals correspond to ± 1 standard deviation.

When statistically comparing the sham and real TMS responses, condition 3 reveals a pattern consistent with TEPs (Fig. 2C) as has been described earlier^13^, with a positive deflection over the stimulated sensorimotor cortex at 30 ms (P30; sensor space: *p* = 0.034, source space: *p* = 0.019), followed by a negative deflection at 45 ms (N45; sensor space: *p* = 0.006, source space: *p* = 0.035) and a positive deflection at 70 ms over premotor areas (slightly later than the expected P60; sensor space: *p* = 0.009, source space: *p* = 0.025). Moreover, the source estimation also suggests a positive deflection over the stimulated sensorimotor cortex at around 120 ms (P120; *p* = 0.003).

The statistical comparison of the sham and real TMS responses from condition 1 also reveals the positive deflection at 70 ms over premotor areas on both the sensor (*p* = 0.002) and source spaces (*p* = 0.002). The P30 could only be distinguished on the sensor space (*p* = 0.017), and the N45 only in the source space (*p* = 0.024). A significant difference was also observed between 140 and 200 ms around central regions (sensor space: *p* = 0.018, source space: *p* = 0.009). This can also be seen in the sensor space in condition 2 (*p* = 0.002), and in condition 4 (*p* = 0.006). Also, condition 2 only revealed the P30 (sensor space: *p* = 0.002, source space: *p* = 0.02), and possibly a P120 in the source space (*p* = 0.002). Condition 4 revealed the P30 (sensor space: *p* = 0.006, source space: *p* = 0.025) and N45 (sensor space: *p* = 0.003, source space: *p* = 0.016), but not the P60. It might also have detected a P120, however, this might have been compromised by the PEP mismatch, evidenced by the significant difference around 200 ms on frontocentral regions.

### Real vs. Sham TMS of SMA

Targeting SMA using real and sham TMS elicited a similar N100/P200 complex in all conditions (Fig. 3, Supplement Figure S2), as was the case for M1 stimulation, consistent with PEPs. Additionally, real TMS, but not sham TMS, elicited a clear positive deflection around 55 ms after the stimulus, centered over the stimulated SMA.

The statistical comparison of the sham and real TMS responses from all conditions (Fig. 3) confirmed a significant cluster representing a positive deflection peaking at 55 ms, centered over the stimulated left SMA (sensor level: Cond1 *p* = 0.016, Cond2 *p* = 0.001, Cond3 *p* < 0.001, Cond4 *p* = 0.009; source level: Cond1 *p* = 0.016, Cond2 *p* = 0.002, Cond3 *p* < 0.001, Cond4 *p* = 0.007). A positive deflection at around 25 ms over the ipsilateral sensorimotor cortex was observed in conditions 1 and 2 at both sensor (Cond1 *p* = 0.004, Cond2 *p* = 0.009) and source levels (Cond1 *p* = 0.026, Cond2 *p* = 0.001), but only at sensor level in condition 3 (*p* = 0.009) and at source level in condition 4 (*p* = 0.022). A lateral posterior cluster representing a negative deflection peaking at 40 ms was only observed at sensor level in conditions 3 and 4 (Cond3 *p* = 0.015, Cond4 *p* = 0.005). Significant clusters at around 120 ms on frontocentral regions in conditions 2 and 3 might represent a potential akin to the P120 from M1 stimulation (sensor space: Cond2 *p* < 0.001, Cond3 *p* = 0.005; source space: Cond2 *p* < 0.001, Cond3 *p* = 0.012).

### Comparing the use of different Sham procedures

**Fig. 4:**
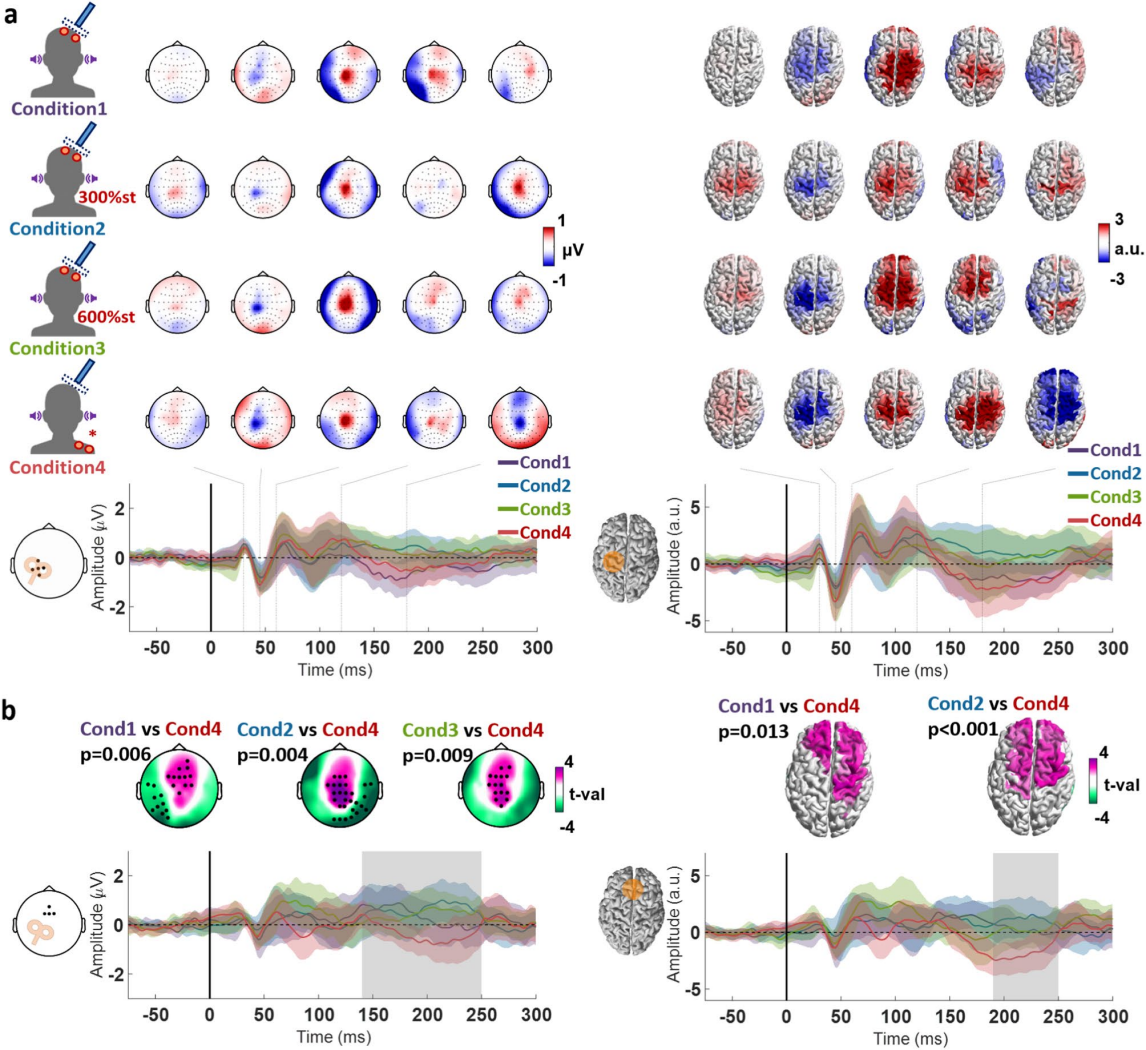
**a.** TMS-EEG after subtraction of the sham response from the real TMS response (TEP) to left M1 stimulation of conditions 1–4. The topographical plots display the signal distribution of the TEP at the following timepoints after stimulation: 30 ms, 45 ms, 60 ms, 120 ms, and 180 ms. Accordingly, the cortical models to the right show the estimated sources of the signals. The time course plots below show the signals of the responses to real minus sham TMS, averaged from electrodes C3, C1, C5, FC3 and CP3 (centered on M1), as illustrated by the corresponding inlet to the left, and on the right, averaged from the dipoles in the area marked in orange in the cortical model to the left. The different colors indicate the 4 conditions, and shaded areas around the time course signals correspond to ± 1 standard deviation. **b.** Statistical comparison of the TEP across the 4 conditions. The topographical plots display the t-value distribution from the post hoc t-tests performed in the time window where the cluster-based ANOVA indicated a significant cluster, marked by a shaded gray area on the time course plot below. The time course plots show the averaged signals from electrodes Fz, F1, F2, AFz (constituting the intersection between the significant clusters). The shaded areas around the time course signals correspond to ± 1 standard deviation. The cortical models to the right show the results from the same analyses performed in the source space, with the time course plots corresponding to the averaged signal from the dipoles in the area marked in orange in the cortical model to the left.

After subtraction of the EEG response to the condition-specific sham TMS from the EEG response to the corresponding real TMS, we compared the TEPs between conditions 1–4 when delivering stimuli to M1. The cluster-based ANOVA revealed a single time window containing a significant difference, in the sensor space between 140 and 250 ms (Cond1 vs. Cond4 *p* = 0.006; Cond2 vs. Cond4 *p* = 0.004; Cond3 vs. Cond4 *p* = 0.009), and in the source space between 190 and 250 ms (Cond1 vs. Cond4 *p* = 0.013; Cond2 vs. Cond4 *p* < 0.001). The post hoc t-tests indicate a consistent difference between condition 4 and the other conditions, represented by a cluster involving frontocentral regions, in both sensor and source space. No other significant difference across conditions was found (Fig. 4).

**Fig. 5:**
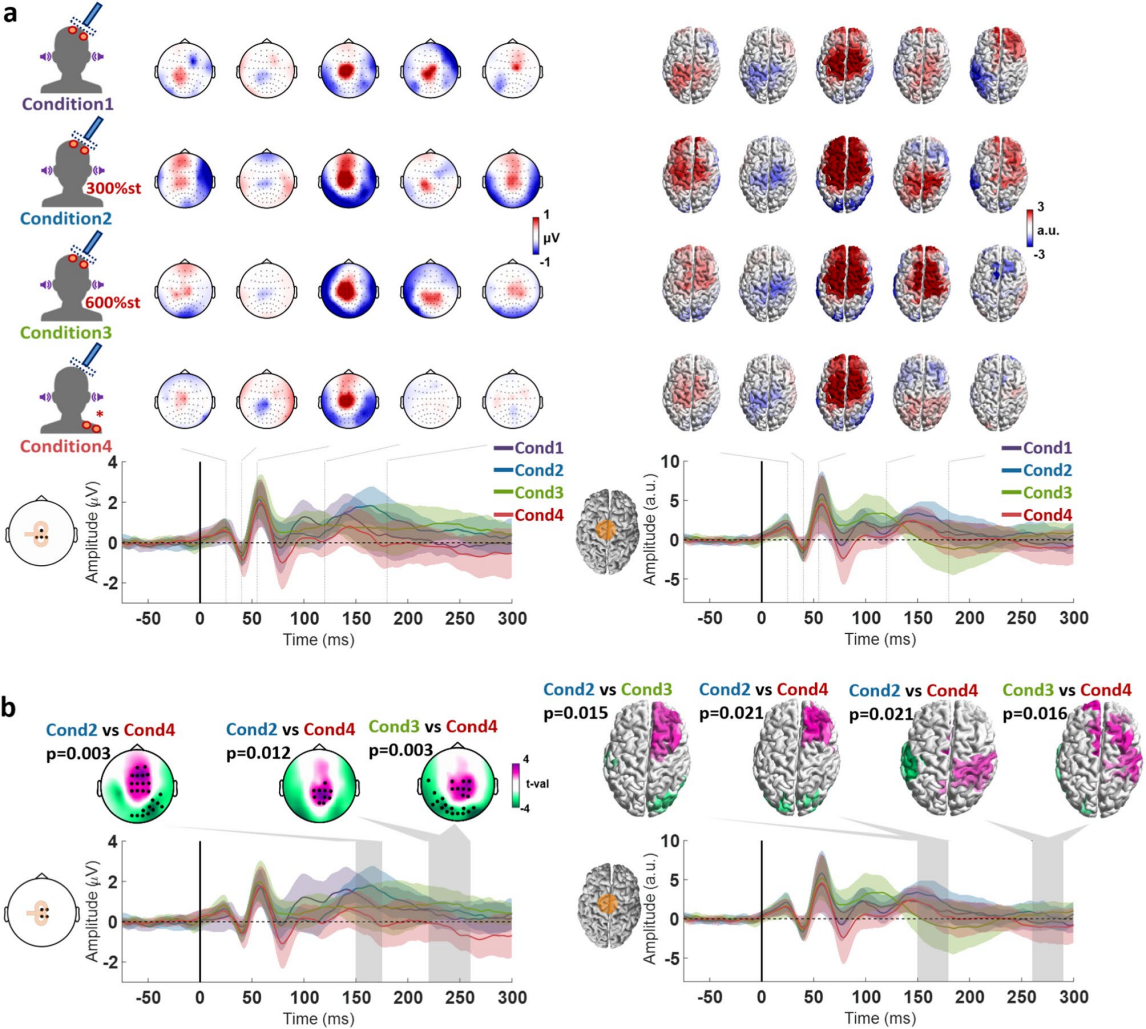
**a.** TMS-EEG after subtraction of the sham response from the real TMS response (TEP) to left SMA stimulation of conditions 1–4. The topographical plots display the signal distribution of the TEP at the following timepoints after the stimulus: 25 ms, 40 ms, 55 ms, 120 ms, and 180 ms. Accordingly, the cortical models to the right show the estimated sources of the signals. The time course plots below show the signals of the responses to real minus sham, averaged from the electrodes Cz, C1, C2 and, FCz (centered on SMA), as illustrated by the corresponding inlet to the left, and on the right, averaged from the dipoles in the area marked in orange in the cortical model to the left. The different colors indicate the 4 conditions, and shaded areas around the time course signals correspond to ± 1 standard deviation. **b**. Statistical comparison of the TEP across the 4 conditions. The topographical plots display the t-value distribution from the post hoc t-tests performed in the time window where the cluster-based ANOVA indicated a significant cluster, marked by the shaded gray areas on the time course plot below. The time course plots show the averaged signals from electrodes FCz, Cz, FC2, C2 (constituting the intersection between the significant clusters). The cortical models to the right show the results from the same analyses performed in the source space, with the time course plots corresponding to the averaged signal from the dipoles in the area marked in orange in the cortical model to the left. The different colors indicate the 4 conditions, and shaded areas around the time course signals correspond to ± 1 standard deviation.

When comparing the TEP from each condition following SMA stimulation, the cluster-based ANOVA revealed 2 time windows with a significant difference, in the sensor space between 150 and 175 ms and 220–260 ms, and in the source space between 150 and 180 ms and 260–290 ms. The post hoc t-tests in the first time window suggest a significant difference between condition 2 and conditions 3 and 4 (sensor space: Cond2 vs. Cond4 *p* = 0.003; source space: Cond2 vs. Cond3 *p* = 0.015, Cond2 vs. Cond4 *p* = 0.021), centered in the right frontal region. The post hoc t-tests of the second time window indicate a difference between condition 4 and conditions 2 and 3 (sensor space: Cond2 vs. Cond4 *p* = 0.012, Cond3 vs. Cond4 *p* = 0.003; source space: Cond2 vs. Cond4 *p* = 0.021; Cond3 vs. Cond4 *p* = 0.016), centered in the sensorimotor cortex contralateral to the stimulation (Fig. 5).

### Equivalence testing of TMS-EEG

**Fig. 6:**
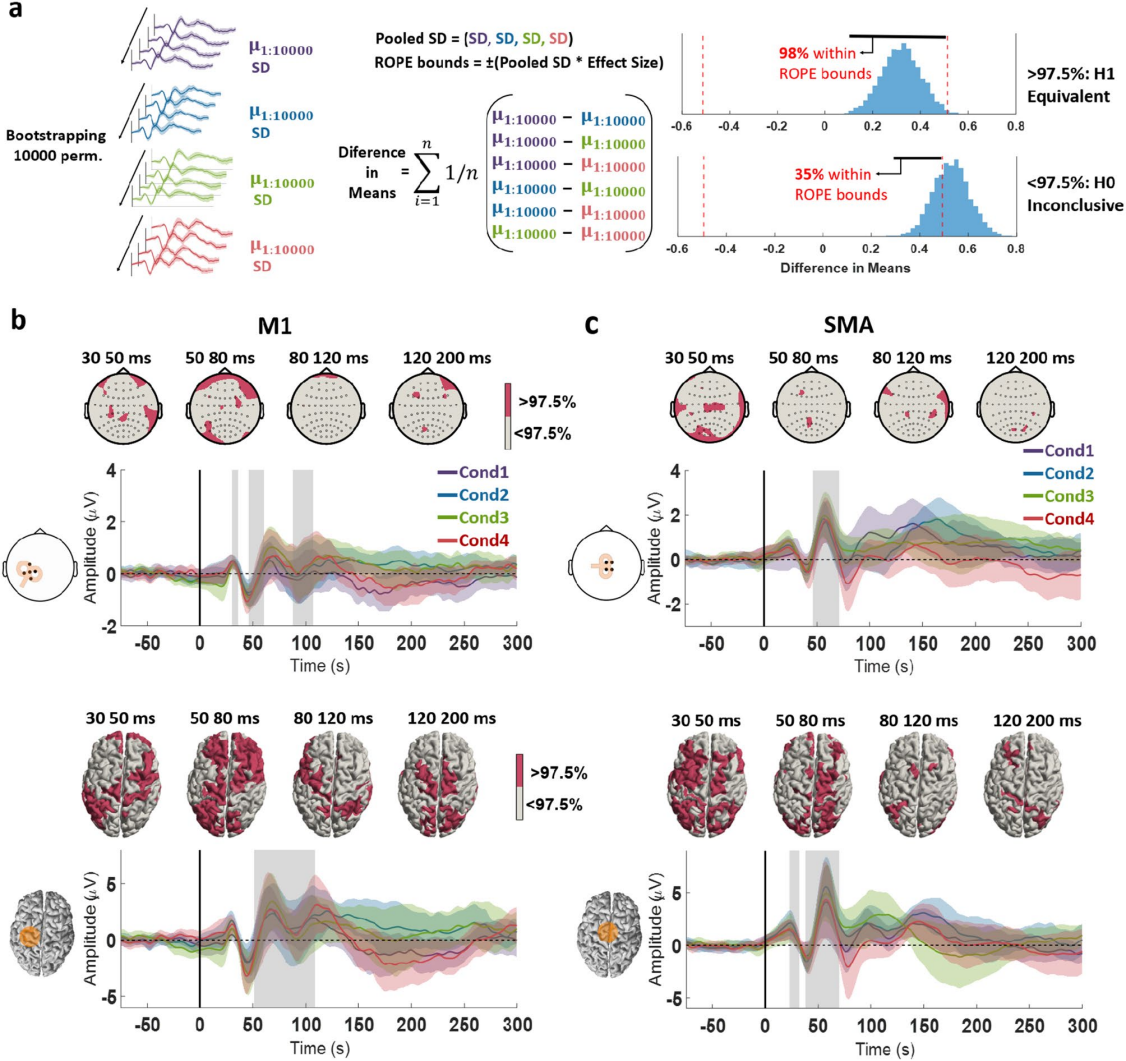
**a**. Diagram illustrating the equivalence test employed. A bootstrapping procedure was used to permute across subjects within each condition, yielding an estimated standard deviation (SD) and a mean (µ) across all subjects for each permutation. The difference in means was computed by subtracting the µ values of the conditions and averaging the result across permutations, resulting in a distribution with the same size as the number of permutations. Equivalence was assessed by calculating the proportion of this distribution of mean differences that fell within the Region of Practical Equivalence (ROPE), defined by the pooled SD and an effect size threshold of 0.4 (Cohen’s d). If more than 97.5% of the distribution lay within the ROPE bounds, the conditions were considered equivalent; otherwise, the null hypothesis (H₀)—that there was no equivalence—could not be rejected. Results of the equivalence testing of the TMS-EEG after subtraction of the sham response from the real TMS response, divided into the responses from M1 (**b**) and SMA stimulation (**c**). The topographical plots and the cortical models display the distribution of the equivalence test significance across all the conditions (p-values corresponding to the highest value obtained in the analysis). Below the time course plots of the EEG response signal of the 4 conditions are displayed, indicated by different colors (averaged across the electrodes in the sensor space analysis marked as black dots; and cortical regions in the source space analysis marked in orange), and shaded areas around the time course signals correspond to ± 1 standard deviation. The shaded gray areas correspond to a time windows of statistical significance of the equivalence test (*p* < 0.05) common to all comparisons.

Finally, we tested the equivalence of the “true” TMS-EEG responses across all four experimental conditions. In the sensor space analysis, only isolated regions showed localized equivalence across conditions. Analysis of the time courses from the stimulated regions revealed significant equivalence around 30, 50 and 110 ms following M1 stimulation and around 30 and 55 ms following SMA stimulation (Fig. 6).

The source space analysis yielded more robust results. For M1 stimulation, we observed high equivalence in the motor cortices during the early time window (30–50 ms), corresponding to the N45 component in this time course. This was followed by strong equivalence also in motor cortices and frontal regions between 50 and 80 ms, aligning with the P60 component, and finally equivalence in the stimulated M1 in the 90–110 ms window. For SMA stimulation, source-level analysis showed the strongest equivalence in central cortical regions surrounding the SMA as well as the left motor cortex within the early time window (30–50 ms). This corresponded to a negative deflection around 40 ms, followed by a positive deflection around 55 ms (Fig. 6).

### Differences between TEPs from M1 and SMA

The time sequence and cortical locations of signal deflections from M1 and SMA stimulation follow somewhat similar patterns. Given the anatomical proximity of these targets, it might be that TMS is in fact exciting both regions and the EEG response is the same. To investigate whether the signals are different we merged the TEP from all conditions (as was justified due to absence of difference between conditions in the first 100 ms, cf. Figures 4 and 5) and compared the response signal from M1 and SMA stimulation.

**Fig. 7:**
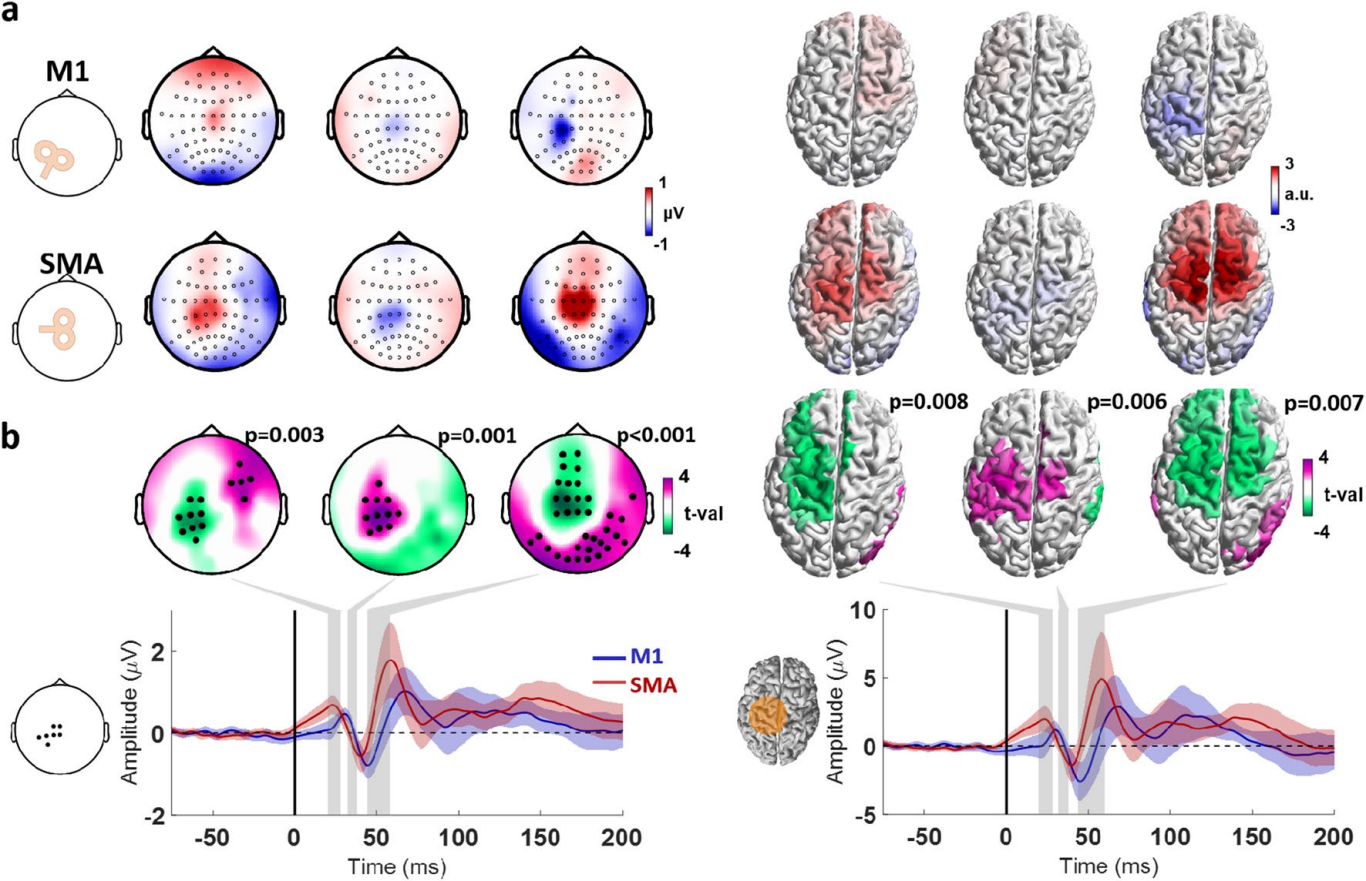
**a.** TMS-EEG after subtraction of the sham response from the real TMS response (TEP) and pooling conditions 1–4, divided into TMS applied to left M1 and left SMA. The topographical plots display the signal distribution of the TEPs at the timepoints where significant clusters were found, marked by the shaded gray areas on the time course plots in **b** Accordingly, the cortical models to the right show the estimated source of the signals. **b**. Statistical comparison of the TEP between M1 and SMA stimulation, with the topographical plots display the t-value distribution. Electrodes composing significant clusters in the sensor space are marked with a dot. The time course plots on the left show the averaged signals from the electrodes highlighted in the inset. The cortical models to the right show the results from the same analyses performed in the source space, with the time course plots corresponding to the averaged signals from the dipoles in the area marked in orange in the cortical model to the left. The different colors indicate M1 stimulation (blue) and SMA stimulation (red), and shaded areas around the time course signals correspond to ± 1 standard deviation.

The cluster-based t-test revealed 3 significant clusters that indicated differences in the TEPs from M1 vs. SMA stimulation, with considerable agreement between the analysis in the sensor and source spaces. The first cluster was between 20 and 30 ms and was centered in the stimulated sensorimotor cortex, likely corresponding to the first positive deflection after SMA stimulation (sensor space *p* = 0.003; source space *p* = 0.008). The second cluster was between 30 and 40 ms and also centered in the stimulated sensorimotor cortex, likely corresponding to an intersection between the M1 P30 and a negative deflection after SMA stimulation (sensor space *p* = 0.001; source space *p* = 0.006). The last cluster was between 40 and 60 ms, centered in the stimulated M1 and adjacent frontocentral regions, likely corresponding to an intersection between the M1 N45 and the SMA positive deflection around 55 ms (sensor space *p* < 0.001; source space *p* = 0.007) (Fig. 7).

## Discussion

In the present study we tested the effects of different sham procedures to unravel the underlying cortical activation by real TMS, i.e., the TMS evoked potentials (TEPs). Specifically, we aimed to test whether the presence of high-intensity somatosensory stimuli in the sham procedures modulates TEPs. We observed that the TEPs obtained from conditions that involved concomitant high-intensity somatosensory stimulation were not significantly different from those without additional sensory input, specifically the responses observed in latencies below 110 ms after TMS. Lastly, in the process of these investigations, we developed another sham method for TMS–EEG, which involves individually calibrating the ES intensity in the sham procedure, ideally resulting in matching the PEPs elicited by sham TMS and real TMS conditions, the PEP individualized matching stimulus intensity calibration (PIMSIC). This method is potentially as effective as our previous sham design of “PEP saturation”, with the advantage of not requiring the use of high-intensity ES during sham and real TMS conditions. We will discuss these results below in more detail and present some observations and limitations.

It is imperative to reliably remove the cortical responses to sensory inputs from the TMS-EEG response signal to obtain the truly specific cortical activation by TMS, the TEPs. This is particularly challenging given the inherent difficulties involved in designing a proper control for PEPs in TMS–EEG experiments^6,9^. To reliably remove PEPs from the TMS–EEG response we applied our previously described “PEP saturation” sham procedure, which involves the delivery of high-intensity somatosensory stimulation via scalp ES to both the sham and real TMS condition, aiming at saturating the cortical response to somatosensory input^13^. In this framework, the saturated PEPs from the sham and real TMS conditions should consistently match, allowing the subtraction of the sham TMS-EEG response from the real TMS-EEG response, thus revealing the TEPs without contamination by PEPs^13,25^. However, a critical point of this procedure is that the high-intensity somatosensory stimulus in the real TMS condition may modulate the response of the brain to TMS. This would render the simple arithmetic subtraction of the sham TMS-EEG response from the real TMS-EEG response erroneous. To address this issue, a procedure that does not involve the delivery of high-intensity somatosensory stimuli during the real TMS condition is necessary. For this purpose, we developed a sham procedure, in which the intensity of the somatosensory stimulus (scalp ES) is individually calibrated so that the resulting PEP amplitude matches that of the PEP amplitude elicited in the real TMS condition without scalp ES^25^. Additionally, we investigated whether delivering somatosensory stimuli to the shoulder would have different effects from delivering ES specifically to the scalp region where TMS is applied.

Regarding the effectiveness of the sham control procedure, we observed that the condition that involved high-intensity scalp ES to both sham and real TMS conditions at 300% of the somatosensory perception threshold presented significant differences in the response signal around 180 ms after the stimulation, in frontocentral regions, when targeting both M1 and SMA (Figs. 2B and 3B). This is likely the consequence of a PEP mismatch between the real and sham conditions. While we used the same stimulation parameters as in our previous report^13^, it is possible that this was not sufficient for some subjects of the present sample to reach the saturation point, resulting in the observed difference. Moreover, by increasing the high-intensity ES in both the sham and real TMS conditions to 600% of the somatosensory perception threshold, these differences were no longer present, suggesting successful saturation and matching of PEPs in the sham and real TMS conditions. This highlights the challenges in designing a proper sham condition for TMS–EEG and the importance of properly determining a sufficiently high ES intensity when using a sham TMS condition according to the “PEP saturation” design.

Regarding the resulting signal from the trials in which PIMSIC was applied, there was no evident difference in the PEPs from sham and real TMS conditions when the ES was applied to the scalp. However, when the ES was applied to the shoulder, there was a significant difference in PEPs between sham and real TMS when the target was M1, but not when the target was the SMA (Fig. 2D). This, however, is likely due to an improper calibration in this particular condition, with the somatosensory stimulus from the sham TMS condition being of higher intensity than the real TMS. Moreover, when targeting the SMA, the N100/P200 complex from the PIMSIC sham to the shoulder precisely matched that from the real TMS. Collectively, this also highlights the importance of properly calibrating the ES intensity for each sham TMS condition, which should not lead to a sensory input that exceeds that of the real TMS condition.

The TEPs from M1-TMS, estimated by subtracting the sham TMS-EEG response from the real TMS-EEG response in each condition, are consistent with previous reports, when stimulating at intensities below MEP threshold, with the typical P30, N45 and P60 TEP components^12,13,55^. We did not observe earlier responses as the signal from time windows < 20 ms was removed due to the massive artifact produced by the high-intensity ES and consequent muscle response. Moreover, the findings also suggested a positive deflection at around 100 and 120 ms over the stimulated sensorimotor cortex. Although we have already found some indication of this response in another experiment^13^, the evidence of this P120 has been inconsistent. One reason is that this hypothetical response is superimposed by the high-amplitude N100/P200 PEP complex, which requires a very reliable sham procedure to reveal eventual TEPs in this time window, which has been lacking in most previous studies^6^. Another issue is that this potential seems specific to subthreshold M1-TMS, as M1-TMS above MEP threshold elicits a negative potential in the stimulated sensorimotor cortex at around this latency^10,37^. TEPs from suprathreshold M1-TMS, however, might be contaminated by cortical responses to reafferent somatosensory feedback from the MEP, a still unresolved issue. The particular sensitivity of this potential to the stimulus intensity might enhance its inter-subject variability, adding to the inconsistent findings.

The TEP obtained from SMA-TMS is also consistent with previous reports, although these are considerably fewer in number compared to reports from M1-TMS^56^. As with the M1-TMS, there was no difference in TMS-EEG responses earlier than 150 ms after stimulation across the tested sham procedures. This further weakens the hypothesis that concomitant somatosensory input modulates TMS–EEG responses. At longer latencies, however, there were 2 significant clusters. The difference at around 150 ms to 180 ms in frontocentral regions is mostly due to increased amplitudes in Condition 2 (Fig. 5), which is likely the result of a PEP mismatch. However, the difference in the response around 250 ms after stimulation between conditions 2 and 3 with condition 4 is unlikely to be attributed to a PEP mismatch, in particular for conditions 3 and 4, as there is no evidence of a N100/P200 mismatch between the responses to real TMS and sham in these conditions (Fig. 3). Moreover, the real TMS was the same in all conditions, which combined should have resulted in identical EEG responses. Therefore, a difference in the EEG response between these conditions suggests a differential interaction effect between the TMS response and the response to somatosensory input across these conditions. Although this latency (250 ms) is considerably later than where typical TEPs are expected, cortical responses to sensory inputs can be observed for hundreds of milliseconds after the stimulus. It is possible that the sensory responses, which are considerably stronger in conditions 2 and 3, are being differentially modulated by SMA direct stimulation by TMS. Moreover, the effects are most pronounced in the sensorimotor cortex contralateral to the side where the somatosensory stimulus was applied, supporting the notion of a modulation of the PEP. The SMA is considered to have an important role in the process of incoming sensory information and generating PEPs^57,58^. It is therefore possible that the sensory input processing in conditions using high-intensity was modulated by the concomitant application of TMS to the SMA.

The main finding of the present study is that no significant differences in TEPs of latencies < 120 ms after stimulation were observed between conditions, regardless of the intensity of the concomitant somatosensory stimulus. This provides strong evidence against the hypothesis that concomitant somatosensory input modulates TMS–EEG responses. The only significant differences were found at longer latencies, namely a high-amplitude deflection from 150 ms up to 250 ms observed in the condition involving PIMSIC with ES applied to the shoulder (Condition 4), likely a consequence of PEP mismatch between the sham and real TMS conditions (Fig. 4). The equivalence analysis further suggests that early TMS-evoked potentials (< 110 ms TEPs) are largely unaffected by incoming sensory stimuli. This was most clearly observed in the source space analysis, which, as previously indicated, likely offers a more reliable representation of TMS-evoked brain activity. Significant equivalence was observed mostly within time windows around 50 and 100 ms, and primarily in signals originating from the stimulated cortical regions (both M1 and SMA). The observed equivalence around 45 ms following M1 stimulation supports the interpretation that the N45 has a component in the contralateral motor cortex, while the P60 is localized to the stimulated hemisphere and frontal regions^55,59^. Furthermore, the consistency of responses around 100 ms in the stimulated cortex lends support to the existence of a late TEP component in this window, typically obscured by peripheral-evoked potentials (PEPs), as discussed earlier. In the case of SMA stimulation, the observed equivalence in the N40 and P55 components reinforces the idea that these early responses are also not modulated by sensory inputs.

Finally, the comparison of TEPs elicited by stimulation of M1 and SMA reveals significant differences in their temporal dynamics and spatial patterns of cortical activation. These findings are consistent with the engagement of distinct neuronal populations and associated circuits, leading to region-specific TEP morphologies even when the targeted areas are only a few centimeters apart^60^. This provides further evidence that TEPs reflect the activation of specific cortical networks in response to TMS.

In summary, the present results suggest that early TMS–EEG responses (< 100 ms) are not sensitive to modulatory effect of sensory inputs delivered at the time of stimulation, even if high-intensity scalp ES is applied in the previously described “optimized sham” procedure^13^. In contrast, sensory stimulation modulates MEP amplitude when applied shortly before TMS, suggesting that it might also modulate TEPs. It was observed that MEP amplitude can be modulated by priming the sensorimotor cortex with incoming somatosensory input, by shifting the attention to the stimuli, or by simply making the subject aware of the incoming TMS pulse^61–64^. In these experiments, somatosensory inputs were delivered prior to the TMS pulse by tens or hundreds of milliseconds, possibly a delay necessary for shifting the sensorimotor cortex excitability state. In contrast, in sham procedures applied here, sensory stimuli and TMS are delivered simultaneously. Therefore, effective TEP modulation by sensory input might be observed with latencies between sensory stimulation and TMS of at least 100 ms^64^. Conversely, we found evidence of an interaction between the TMS response and PEPs at around 250 ms after SMA-TMS. We interpreted this as a modulation of the PEP amplitude due to priming with SMA-TMS, a cortical area relevant for PEP generation.

Another issue is that the continuous delivery of masking noise might have modulated the TEPs. Given the evidence that the presence of continuous white noise modulates MEP amplitudes^65^, it is also possible that this has affected TEP amplitudes in TMS–EEG measurements. Unfortunately, we cannot address this possibility here, as all conditions involved masking noise. However, the available evidence suggests that masking noise in TMS–EEG has no effect on TEPs^12^, which should be confirmed in future experiments. Nevertheless, it should be noted that noise input will always be present in TMS experiments, either in the form of masking noise and/or the TMS pulse.

The results here also demonstrate the feasibility and efficacy of a sham TMS–EEG procedure of individually calibrating ES intensities, PIMSIC. Several previous studies have also reported the use of individually adjusted ES intensity in order to match the individual sensory perception of the real TMS condition^8,23^. This “realistic sham” design requires a thorough match between the multisensory perception from the sham and real TMS conditions^6^. However, this has proven to be considerably challenging, as, despite the best efforts, differences in perceptual qualities between the two conditions can still be reported by at least some individuals^8,13^. Moreover, even in cases where approximate equivalence in perception has been achieved at the group level, as demonstrated by standardized psychophysical questionnaires, a portion of subjects could still distinguish stimuli from the two conditions^13^. Therefore, in the present approach we opted to bypass perceptive reporting, and to focus instead on the neurophysiological response, which is in fact the TMS–EEG endpoint measure. By calibrating ES intensity in the sham condition to match PEP amplitude with the real TMS condition, it assures that the subtraction of the sham TMS-EEG response from the real TMS-EEG response will consistently eliminate the PEPs.

The present study has limitations, mostly inherent to the sham methods themselves. Here we show that the “optimized sham” procedure with high-intensity ES in both the sham and real TMS conditions does not significantly modulate the underlying TEPs, with considerable equivalence between the responses < 110 ms after the pulse. We, therefore, confirm their reliability in matching and removing the PEPs. However, the high-intensity ES applied to the scalp results in a large artifact in the EEG recordings, caused by electrical interference and scalp muscles activation. This considerably compromises signal quality and requires the removal of the first 20 ms of signal after the ES pulse, which may contain relevant information. This might also have compromised the equivalence in early TEPs, such as the P30 following M1 stimulation, which was not significant at source level. A possible solution to this problem could be placing the ES outside the head area and, consequently, away from the EEG electrodes array. We tested this in the present experiment using shoulder ES. Indeed, we found no difference in the PEPs elicited from the shoulder and scalp when using the PIMSIC, which validates previous studies that have used this region as the target for somatosensory input in the sham condition^10,66^. Future studies may consider this region as a valid target in order to avoid placing ES electrodes on the scalp and risking a potentiation of the artifacts in the EEG. Nevertheless, high-intensity somatosensory stimulation may also affect tolerability. Although we did not include a measure of subjective sensory input in the present study, and no participant dropped out or withdrew consent, some participants did report discomfort during high-intensity ES. A more tolerable yet equally reliable approach may involve individualized matching of stimulus intensity, such as with the PIMSIC approach.

The PIMSIC approach also presents limitations in its current form. Our results from M1-TMS with individually calibrated ES to the shoulder as the sham condition reveal a PEP mismatch, indicating a failure of the procedure. Two non-mutually exclusive factors may account for this outcome: first, an insufficient number of sensory stimulation trials resulting in a low signal-to-noise ratio and inaccurate calibration of ES intensity. Second, the amplitude of the PEP may vary through the TMS–EEG session, potentially due to variation in alertness^67^ or physiological fluctuations in top-down mechanisms that modulate attention and sensory responsiveness^68^, leading to a mismatch between the PEPs recorded during calibration vs. those during the main experiment. This issue may be exacerbated by the choice to use late evoked potentials (200–300 ms post-stimulus) as the calibration reference, as these are likely to reflect cognitive responses that are particularly sensitive to internal state fluctuations. In summary, although the PIMSIC approach may represent a valuable alternative to the high-intensity ES “optimized sham” method, it requires further refinement. Specifically, improvements should address the dynamic nature of PEPs over time, reassess the appropriateness of the P200–N300 amplitude as a calibration reference, and incorporate a larger number of sensory stimulation trials to enhance reliability.

## Conclusions

We observed no evidence that TEPs are modulated by concomitant somatosensory inputs within 110 ms post-stimulation. This validates our previous method of controlling for PEPs by using high-intensity scalp ES in both sham and real TMS to thoroughly remove PEPs in TMS–EEG measurements. The present findings also validate effective removal of PEPs from the EEG signal by subtraction of the sham from the real TMS-EEG response, which critically assumes a linear superposition of PEPs and TEPs. Finally, we validated another alternative sham method that involves individual calibration of the somatosensory input in order to generate PEPs equivalent to those elicited by real TMS, which can be applied both on the scalp and on the shoulder, thus exempting the need for high-intensity ES in regions close to the EEG channels, which may cause subject discomfort and interfere with EEG signal acquisition.

## Supplementary Information

Below is the link to the electronic supplementary material.

Supplementary Material 1

## Data Availability

The datasets generated and analyzed during the current study are available from the corresponding author upon reasonable request. The analysis code is publicly available on GitHub: [https://github.com/pcgordon/optimized\_supraliminal\_sham](https:/github.com/pcgordon/optimized_supraliminal_sham) . The codes were designed for MATLAB version 2021b and using the open-source toolbox Fieldtrip, version 20210212.
