# Supplementary material for: Investigating the effects of TMS-related somatosensory inputs on TMS-evoked potentials provides evidence against significant interaction
Source: Sci Rep. 2026 Jan 30;16:4317. doi: 10.1038/s41598-026-37418-w (PMC12865020; doi:10.1038/s41598-026-37418-w)
Supplement: Supplementary file 1 — Supplementary Material 1 [file 41598_2026_37418_MOESM1_ESM.docx]

**Supplement**

**Investigating the effects of TMS-related somatosensory inputs on TMS-evoked potentials: Evidence against significant interaction**

Pedro C. Gordon ^1,2^, Johanna Metsomaa ^3,4^, Paolo Belardinelli ^1,2,5^, Ulf Ziemann ^1,2^*

^1^ Department of Neurology & Stroke, University of Tübingen, Germany

^2^ Hertie Institute for Clinical Brain Research, University of Tübingen, Germany

^3^ Department of Neuroscience and Biomedical Engineering, Aalto University, Espoo, Finland

^4^ BioMag Laboratory, HUS Medical Imaging Center, Helsinki University Hospital, Helsinki University and Aalto University School of Science, Helsinki, Finland

^5^ Center for Mind/Brain Sciences—CIMeC, University of Trento, I-38123 Trento, Italy

| 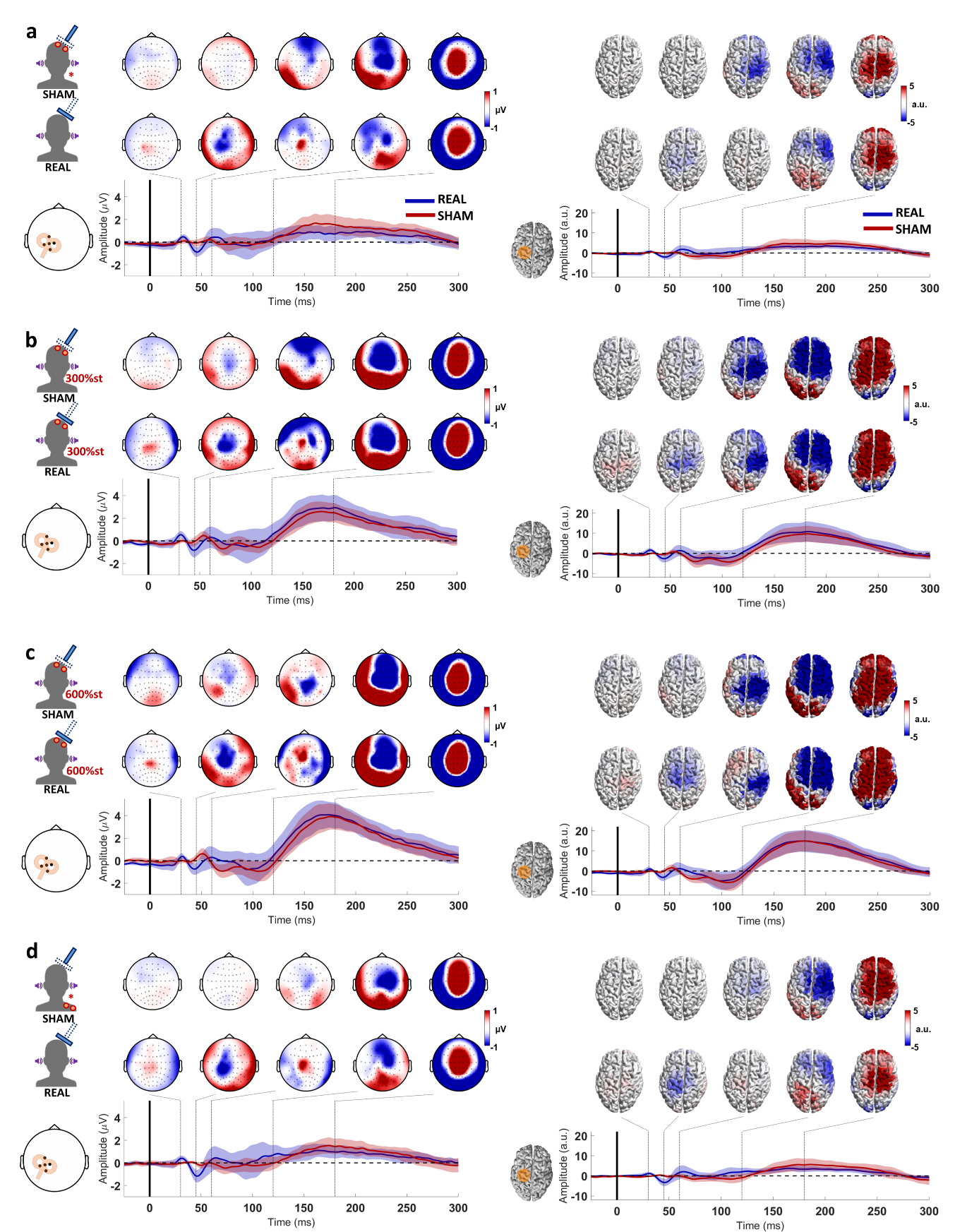 |
| --- |
| **Figure S1.** TMS-EEG responses to left M1 stimulation from each condition, averaged across all subjects: **a.** Condition 1: Individually titrated ES to the scalp only applied in the sham TMS. **b.** Condition 2: ES to the scalp at an intensity of 300% the individual’s somatosensory perception threshold, applied in both the sham and real TMS. **c.** Condition 3: ES to the scalp at an intensity of 600% the individual’s somatosensory perception threshold, applied in both the sham and real TMS. **d.** Condition 4: Individually titrated ES to the left shoulder only applied in the sham TMS.  The topographical plots display the signal distribution of the response to sham TMS (top) and to real TMS (bottom) at the following timepoints after stimulation: 30 ms, 45 ms, 60 ms, 120 ms, and 180 ms. Accordingly, the cortical models to the right show the estimated sources of the signals.  The time course plots below the topographical plots show the signals of the responses to real TMS (blue) and sham TMS (red). On the left, the signals were averaged from electrodes C3, C1, C5, FC3 and CP3, as illustrated by the corresponding inlet to the left; and on the right, signals were averaged from the dipoles in the area marked in orange in the cortical model on the left. The shaded areas around the time course signals correspond to ±1 standard deviation. |

| 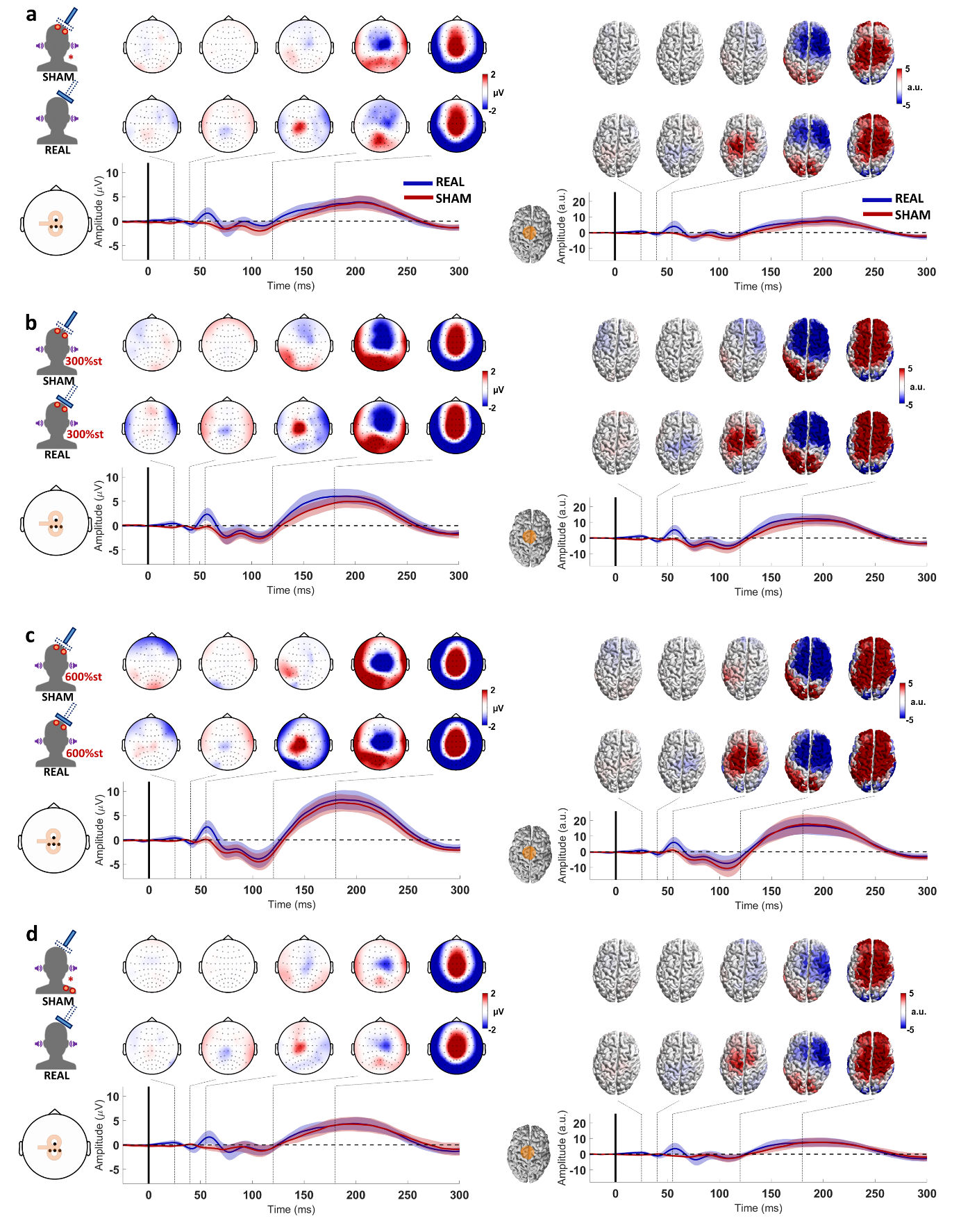 |
| --- |
| **Figure S2.** TMS-EEG response to left SMA stimulation from each condition, averaged across all subjects: **a-d.** Conditions 1-4 as described in Figure 2.  The topographical plots display the signal distribution of the response to sham TMS (top) and to real TMS (bottom) at the following timepoints after the stimulus: 25 ms, 40 ms, 55 ms, 120 ms, and 180 ms. Accordingly, the cortical models to the right show the estimated sources of the signals.  The time course plots below the topographical plots show the signals of the responses to real TMS (blue) and sham TMS (red). On the left, averaged from the electrodes Cz, C1, C2 and, FCz, as illustrated by the corresponding inlet to the left; and on the right, averaged from the dipoles in the area marked in orange in the cortical model to the left. The shaded areas around the time course signals correspond to ±1 standard deviation. |
